# Estimating the Readily-Releasable Vesicle Pool Size at Synaptic Connections in the Neocortex

**DOI:** 10.3389/fnsyn.2019.00029

**Published:** 2019-10-15

**Authors:** Natalí Barros-Zulaica, John Rahmon, Giuseppe Chindemi, Rodrigo Perin, Henry Markram, Eilif Muller, Srikanth Ramaswamy

**Affiliations:** ^1^Blue Brain Project, École Polytechnique Fédérale de Lausanne, Geneva, Switzerland; ^2^Laboratory of Neural Microcircuitry, Brain Mind Institute, École Polytechnique Fédérale de Lausanne, Lausanne, Switzerland

**Keywords:** synaptic transmission, quantal analysis, multi vesicular release, neocortex, mathematical model, short-term depression

## Abstract

Previous studies based on the ‘Quantal Model’ for synaptic transmission suggest that neurotransmitter release is mediated by a single release site at individual synaptic contacts in the neocortex. However, recent studies seem to contradict this hypothesis and indicate that multi-vesicular release (MVR) could better explain the synaptic response variability observed *in vitro*. In this study we present a novel method to estimate the number of release sites per synapse, also known as the size of the readily releasable pool (N_RRP_), from paired whole-cell recordings of connections between layer 5 thick tufted pyramidal cell (L5_TTPC) in the juvenile rat somatosensory cortex. Our approach extends the work of Loebel et al. (2009) by leveraging a recently published data-driven biophysical model of neocortical tissue. Using this approach, we estimated N_RRP_ to be between two to three for synaptic connections between L5_TTPCs. To constrain N_RRP_ values for other connections in the microcircuit, we developed and validated a generalization approach using published data on the coefficient of variation (CV) of the amplitudes of post-synaptic potentials (PSPs) from literature and comparing them against *in silico* experiments. Our study predicts that transmitter release at synaptic connections in the neocortex could be mediated by MVR and provides a data-driven approach to constrain the MVR model parameters in the microcircuit.

## Introduction

Synaptic transmission is the basis for neuronal communication and information processing in the brain. Synaptic communication between neurons is mediated by neurotransmitters contained in presynaptic vesicles that are stochastically released from axonal boutons by incoming action potentials (APs) and diffuse across the synaptic cleft to bind receptors. Synaptic receptors are a class of ion channels which open as a result of transmitter binding, and the resulting transmembrane currents either depolarize or hyperpolarize the postsynaptic membrane, depending on the ion to which the channel is permeable (Mason et al., 1991; Südhof, 2000). Understanding the mechanisms behind vesicle release is crucial to unravel how information propagates between neuron types (Tsodyks and Markram, 1997). Disrupted vesicle release is implicated in pathologies such as Alzheimer’s disease or schizophrenia (Waites and Garner, 2011).

In 1954, del Castillo and Katz described the ‘Quantal model’ of synaptic transmission (Del Castillo and Katz, 1954). This model is characterized by the number of independent release sites (N), the probability of releasing a vesicle in the presynaptic cell followed by an AP (p) and the content of each vesicle, the quantal size (q), which collectively determine the efficacy of synaptic transmission (Del Castillo and Katz, 1954; Tsodyks and Markram, 1997). Previously, it was thought that no more than one vesicle could be released per synaptic contact, leading to the univesicular release hypothesis (UVR), in which N is equal to the number of physical synaptic contacts in a neuronal connection, at least for synapses in the neocortex (Korn et al., 1981, 1994; Silver et al., 2003; Biró et al., 2005). However, evidences as fluctuations of evoked postsynaptic potentials (PSPs) (Tang et al., 1994), large concentration of neurotransmitter in the synaptic cleft (Tong and Jahr, 1994) or a high range variability of receptor-mediated signals of *N*-methyl-D-aspartate (NMDA) and α-amino-3-hydroxy-5-methyl-4-isoxazolepropionic acid (AMPA) receptors (Conti and Lisman, 2003) suggested that transmission at a single synaptic contact could be multiquantal. Consequently, a multivesicular release hypothesis (MVR) was proposed, where several release sites could underlie a synaptic contact in a neuronal connection. In fact, there are evidences showing that MVR occurs in brain regions such as the hippocampus (Tong and Jahr, 1994; Christie and Jahr, 2006), the cerebellum (Auger et al., 1998), the hypothalamus (Gordon, 2005) or the cerebral cortex (Huang et al., 2010; Rudolph et al., 2015; Molnár et al., 2016).

Recent studies in the rodent neocortex support the idea of MVR between pyramidal cells (Loebel et al., 2009; Hardingham et al., 2010; Rollenhagen et al., 2018). It has also been reported that modalities of vesicle release differ across cortical areas. For instance, connections between excitatory neurons in layer 4 exhibit UVR in the primary visual cortex, as against MVR in the primary somatosensory cortex (Huang et al., 2010). By contrast, other studies have reported that connections between layer 4 stellate cells and layer 2/3 pyramidal cells in the rat barrel cortex (Silver et al., 2003), and between pyramidal cells and interneurons in the rat cortex (Molnár et al., 2016) display UVR. A recent study has also reported that connections between pyramidal cells and fast spiking interneurons in the human neocortex exhibit MVR (Molnár et al., 2016). MVR is a complex process that is thought to regulate synaptic transmission and plasticity by increasing the dynamic range of synapses and could, therefore, influence cognitive functions such as learning and memory (Fuhrmann et al., 2004). MVR is also known to directly impact synaptic noise through spontaneous miniature postsynaptic currents (Fatt and Katz, 1950) and synaptic variability resulting in an increase of synaptic strength through larger vesicle pool sizes (Oertner et al., 2002), which could have important implications in the transmission of information between neurons (Fuhrmann et al., 2004).

Theoretical and computational models have enabled a mechanistic understanding of MVR through investigating synaptic processes such as short-term synaptic plasticity (Hennig, 2013). These models account for parameters to model presynaptic processes including the probability of neurotransmitter release and the number of vesicles available for release (Tsodyks and Markram, 1997; Loebel et al., 2009; Hennig, 2013; Zhang and Peskin, 2015). In addition, these models also assume that each synaptic contact has access to a limited amount of releasable neurotransmitter, take into account vesicle depletion and replenishment (Liley and North, 1953), and facilitation mechanisms (Betz, 1970; Varela et al., 1997; Markram et al., 1998). Some models have also demonstrated an important functional role for the number of release sites per synaptic connection in neuronal information coding (Fuhrmann et al., 2002). It has also been reported that the number and frequency of vesicles released is essential for receptor activation (Boucher et al., 2010). Some studies also outline the importance of having a readily releasable pool (N_RRP_) with more than one vesicle for synaptic plasticity (Nadkarni et al., 2010). Despite the importance of MVR in information transmission and processing between neurons, we lack an understanding of its role in brain regions such as the neocortex, which is the seat of higher order cognitive functions in the mammalian brain.

In this study, we leveraged a rigorously validated data-driven model of neocortical tissue at the cellular and synaptic levels of detail to estimate the average size of the N_RRP_ for individual synaptic contacts between cell-type-specific connections (Markram et al., 2015). To compute the N_RRP_, we sampled synaptically connected pairs of neurons within the virtual neocortical tissue model and simulated paired whole-cell recordings *in silico*. The properties of *in silico* synaptic connections were constrained by an experimental dataset that characterized the physiology of *in vitro* synaptic connections between layer 5 thick-tufted pyramidal cells (L5_TTPC) in the juvenile rat somatosensory cortex, which are marked by prominent short-term depression (Ramaswamy and Markram, 2015). In particular, we used this dataset to estimate synaptic noise and the MVR free parameter N_RRP_, extending the work of Loebel and colleges (Loebel et al., 2009). Next, we optimized the N_RRP_, to reproduce response variability as observed in experiments, which is typically assessed by the coefficient of variation (CV; standard deviation/mean) of PSPs. We further developed an approach to estimate N_RRP_ for both excitatory and inhibitory connection types using published literature that reported the CV of PSPs for synaptic connections in the neocortex. Our study combining *in vitro* experiments and *in silico* computational modeling, predicts that the vast majority of synaptic connections in the neocortex are mediated by MVR, albeit with lower N_RRP_ values than previously reported (Loebel et al., 2009), which suggests that MVR could be a general property of local neocortical connections.

## Materials and Methods

### Slice Preparation and Electrophysiology

Fourteen- to eighteen-day-old Wistar rats were decapitated according to the guidelines of the Swiss Animal Welfare Act, and the Swiss National Institutional and Veterinary office guidelines in the Canton of Vaud on Animal Experimentation for the ethical use of animals. Multiple, simultaneous somatic whole cell patch-clamp recordings from clusters of 6–12 cells were carried out with Multiclamp 700B amplifiers in current clamp mode. Brain sagittal slices of 300 μM width were cut on an HR2 vibratome (Sigmann Elektronik). Temperature was maintained at 34 ± 1°C in all experiments. The extracellular solution contained 125 mM NaCl, 2.5 mM KCl, 25 mM D-glucose, 25 mM NaHCO_3_, 1.25 mM NaH2PO4, 2 mM CaCl2, and 1 mM MgCl2 bubbled with 95% O2 and 5% CO2. The intracellular pipette solution contained 110 mM potassium gluconate, 10 mM KCl, 4 mM ATP-Mg, 10 mM phosphocreatine, 0.3 mM GTP, 10 Hepes, and 13 mM biocytin adjusted to pH 7.3–7.4 with 5 M KOH.

**FIGURE 1:**
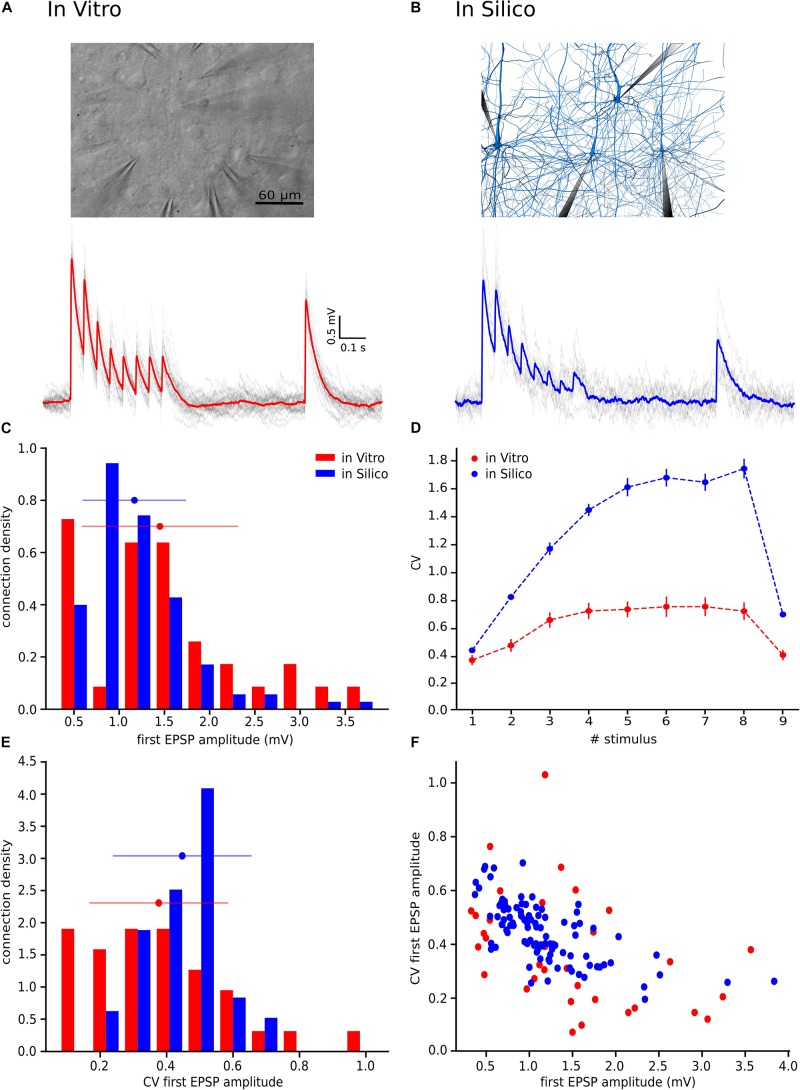
With the UVR hypothesis it was not possible to reproduce the variability observed *in vitro*. **(A)** Example of a multiple whole cell patch-clamp recording in L5_TTPC connections (top). *In vitro* mean voltage trace (bottom; red) of 20 protocol repetitions (gray). **(B)** Illustration of an *in silico* patch-clamp experiment performed on L5_TTPC connections from the data-driven model of the rat cortex microcolumn. *In silico* mean voltage trace (bottom; blue) of 20 protocol repetitions (gray). **(C)** Histogram showing the distribution of the first EPSP amplitude for *in vitro* (red) and for *in silico* (blue) experiments. **(D)** Mean CV profiles for the *in vitro* (red) and the *in silico* (blue) experiments. **(E)** CV distribution of the first EPSP amplitude for *in vitro* (red) and *in silico* (blue) data sets. **(F)** Raster plot of the first EPSP amplitude against the CV of the first EPSP amplitude for *in vitro* (red) and *in silico* (blue) experiments. In the distributions and the CV profile, dots represent the mean and vertical and horizontal bars represent the standard deviation of all the experiments respectively.

Data was acquired through an ITC-1600 board (Instrutech) connected to a PC running a custom-written routine (PulseQ) under IGOR Pro (WaveMetrics, Lake Oswego, OR, United States). L5_TTPCs were selected according to their large soma size (15–25 μm) and their apparent large trunk of the apical dendrite. Cells were visualized by infrared differential interference contrast video microscopy using a VX55 camera (Till Photonics) mounted on an upright BX51WI microscope (Olympus). Sampling rates were 5–10 kHz, and the voltage signal was filtered with a 2-kHz Bessel filter. The resting membrane potential was −65.3 ± 4.3 mV, the input resistance was 59.7 ± 17.1 MΩ and the access resistance was 15.2 ± 3.7 MΩ. The stimulation protocol consisted of pre-synaptic stimulation with eight electric pulses at 20 Hz followed by a single pulse 500 ms later (recovery test), at the sufficient current intensity to generate APs in the presynaptic neuron while the postsynaptic neuron responses were recorded. The protocol was repeated between 20 to 60 times with a time between repetitions of 12 s (Figure 1A, top).

### Stochastic Model for Short-Term Dynamics and Multi-Vesicular Release

Our model describes the short-term synaptic dynamics defined by a stochastic generalization of the Tsodyks-Markram model (TM-model) (Tsodyks and Markram, 1997; Maass and Markram, 2002) that is known to fit excitatory as well as inhibitory synapses behavior of biological experiments (Markram et al., 1998; Gupta et al., 2000). This model considers that there is a finite number of vesicles ready to be released defined by N_RRP_ that could be in ready or recovery state. In this study we followed the synaptic dynamics described previously that is able to predict the sequence of PSP amplitudes produced by any spike train (Tsodyks and Markram, 1997). This behavior is described by four main synaptic parameters: the absolute synaptic efficacy (*A*), the fraction of synaptic resources used by a single spike (*U*), the time constant for recovery from facilitation (*F*) and the time constant for recovery from depression (*D*). The PSP amplitudes prediction obeys the following mathematical expressions:

$$A_{n}=A\,u_{n}\,R_{n}$$

A=1

$$u_{1}=U$$

$$R_{1}=1$$

$$u_{n+1}=U+u_{n}\,\left(1-U\right)\,\exp\left(-\frac{\Delta \,t_{n}}{F}\right)$$

$$R_{n+1}=1+\left(R_{n}-R_{n}\,u_{n}-1\right)\,\text{exp}\,\left(-\frac{\Delta \,t_{n}}{D}\right)$$

In short, when the *n*th spike occurs there is certain fraction of synaptic efficacy modeled by *R*_n_. Accordingly, the product *u*_n_*R*_n_ models the fraction of synaptic efficacy used by the *n*th spike. Combining these terms is possible to describe the fraction of synaptic efficacy available when the next spike arrives at time Δ*t*_n_ assuming that the synaptic efficacy has an exponential recovery with time constant *D*. How much fraction of synaptic efficacy (*R*_n+1_) is used when (*n* + 1)th spike occurs is defined by *u*_n+1_ which increases for each subsequent spike from *u*_n_ to *U*(1–*u*_n_) + *u*_n_ and goes back to *U* following an exponential with time constant *F* (Maass and Markram, 2002).

Thus, if a vesicle is successfully released, these receptors get activated with a conductance g_max_/N_RRP_ with g_max_ as the maximal conductance.

### Fitting Synapse Model Parameters to the Data

We constrained our synaptic model by extracting the parameters *U*, *D* and *F* from *in vitro* connections (*n* = 33; Figures 3C–E). To this end, we measured the peak for the excitatory postsynaptic potential (EPSP) amplitudes of each averaged voltage trace (Figure 3A). All experimental traces were normalized to their maximum value that allowed us to directly compute the peak value instead of the total amplitude. To perform an accurate computation of the peaks we used an analytical tool for deconvolving the voltage averaged trace (Richardson and Silberberg, 2008), which made it possible to exclude the smoothing effect of the low pass filtering of the cell membrane with a time scale equal to τ_mem_, so we could extract the peaks from the EPSPs (Figure 3B).

To express this process mathematically we used the next equation:

$$R_{i\,n\,p\,u\,t}\,I_{s\,y\,n}=\tau_{m\,e\,m}\,\frac{d\,V}{d\,t}+V$$

The right-hand part of the expression is the voltage deconvolution, while the left hand contains the unfiltered synaptic current. The requirement here is to compute τ_mem_ for each *in vitro* connection by fitting the decay part of the recovery peak (9th EPSP) of the averaged voltage trace to an exponential.

Once the EPSP peaks were extracted from the deconvolved and normalized trace, we introduced them as an input into a genetic algorithm (GA) (Goldberg and Holland, 1988) that creates 500 generations of potential *U*, *D* and *F* within the following ranges *U* (0–1.0), *D* (0–1000.0) and *F* (0–2000.0). According to the mathematical expression of the model, the GA was able to estimate the peaks per different generation. The GA minimized the mean square distance between the original and the estimated peaks giving one solution for the minimum distance. In order to optimize the result, the GA was run 50 times. Then, we considered that the *U*, *D*, *F* generation related with the minimum distance out of the 50 repetitions was the best solution. We performed that process for each of the *in vitro* connections.

### *In silico* Experiments: The Cortical Microcircuit

For the *in silico* experiments we leveraged a previously published model of juvenile rat somatosensory cortical tissue (Markram et al., 2015). In brief, the tissue model consists of 31,000 morphologically detailed neurons distributed across 6 layers within a volume of 0.29 mm^3^ giving rise to 8 million synaptic connections mediated by 37 million synaptic contacts. All the neuronal and synaptic models can be freely obtained through the open-access Neocortical Microcircuit Collaboration (NMC) portal (Ramaswamy et al., 2015).

Having computed the mean and the standard deviation of the synaptic parameters from fitting the *in vitro* data to the TM-model, we updated these parameters in the neocortical tissue model that were implemented as distributions defined by their mean and standard deviation. We also computed by scaling its values until we matched the experimentally measured amplitude of the first EPSP in a train of responses, which determined the *in silico* g_max_ value for a simulated connection. Next, we performed patch-clamp *in silico* experiments (Figure 1B, up), under similar conditions to actual *in vitro* paired recordings, with different N_RRP_ values. These values were defined based on the mean of a Poisson distribution shifted one unit to the right, because at least one vesicle had to be released per synaptic site. The range of means of the Poisson distributions varied from 0 to 13 (1 ≤ N_RRP_ ≥ 14) in the case of studying MVR and 0 (N_RRP_ = 1) while studying UVR. We decided to set the maximum value to 14 vesicles on average per release site because is already the double of what Loebel and colleges predicted on their research (Loebel et al., 2009), so we considered that no more than 14 vesicles could be released per synaptic contact.

As the next step, we simulated 100 L5_TTPC connections *in silico* with 20 stimulus-response repetitions each. To reproduce *in vitro* experiments as faithfully as possible, we ensured that the U, D and F distributions *in silico* were identical to those extracted from *in vitro* recordings. We then compared the resulting EPSPs of simulated *in silico* connections to ascertain that they were well the range of experimentally measured values, consequently the EPSPs out of the experimental range were eliminated. Therefore, we excluded 15 connections and undertook the study with 85 connections out of 100.

### Noise Calibration. Ornstein-Uhlenbeck Process

After simulating *in silico* connections with different N_RRP_ values and selecting a subset where the 1st EPSP amplitude was within the experimentally observed range, we artificially applied voltage fluctuations to *in silico* traces to take into account the membrane noise observed experimentally. This was achieved by implementing an Ornstein-Uhlenbeck process (OU-process), which is a stochastic process that allowed us to simulate small random variability. The OU-process describes the velocity of the movement of a Brownian particle considering the friction and is a stationary Gauss-Markov process (Enrico Bibbona, 2008).

Mathematically the expression used in this work for this process was:

$$X\,\left(t+1\right)=X\,\left(t\right)-\frac{X\,\left(t\right)}{\tau }\,d\,t+\sigma \,\sqrt{\frac{2}{\tau }\,d\,t}\,\,W_{t}$$

$$X\,\left(t_{0}\right)=\,x_{0}$$

Where τ is the membrane time constant, σ is the standard deviation of the voltage and W_t_ is a random term coming from the Wiener process. In the case of σ = 0 the equation will have the solution *X*(*t*) = *x*_0_*e*^−(*t*−*t*_0_)/τ^ so X(t) relaxes exponentially toward 0. In general, X(t) fluctuates randomly, the third term pushes it away from zero, while the second term pulls it back to zero (Bibbona et al., 2008). In Physics this process is used to describe noisy relaxation activity.

In our specific case, we defined σ and τ using the voltage values between the 8th and the 9th EPSPs, 400 ms in total, for each repetition (sweep) in a connection and then we averaged the resulting values (Figure 4A). By computing the standard deviation of these points, we obtained one σ per connection (*n* = 33 connections in total). By computing the autocorrelation of this part of the voltage trace and fitting it to an exponential, we obtained one τ per connection (Figure 4B), which provided constraints to implement a similar membrane noise for *in silico* traces (Figure 4C).

### CV Profile Computation. The Jack-Knife Bootstrapping Analysis

In order to compute the CV for the EPSP amplitudes for *in vitro* and *in silico* connections in a comparable way, we implemented the Jack-Knife method (JKK) (Efron and Tibshirani, 1994).

This method consists in excluding one observation at a time from a group of observations. In our specific case, from a set of single traces we computed the average of all but one off the traces each time, obtaining a set of averaged-JKK traces in the end. From each of these averaged-JKK traces we computed the amplitudes for all nine EPSPs in a train of synaptic responses. Through this computation, we were able to compute the EPSP amplitudes more precisely considering that we removed the noise by averaging. Thereafter, we computed the CV profiles for the *in vitro* data set and the *in silico* simulations using the following equations:

$$C\,V^{n}=\frac{s\,t\,d^{n}}{{\bar{A}}^{n}}$$

$${\bar{A}}^{n}=\frac{1}{N}\,\sum_{i=1}^{N}A_{i}^{n}$$

$$s\,t\,d^{n}=\sqrt{\left(N-1\right)\,\sum_{i=1}^{n}\left(A_{i}^{n}-{\bar{A}}_{i}^{n}\right)}$$

Where n denotes the EPSP index (*n* = 1–9) and N is the number of single traces per connection.

Having two sets of simulations, to study UVR and MVR, we computed the CV profile of EPSP amplitudes using the JKK approach in both cases and compared them with the CV profile measured in the *in vitro* dataset. The EPSP amplitude was computed as the difference between the minimum value within 50 ms before stimulation time and the maximum value within 300 ms after stimulation time. We computed the mean square distance in order to obtain the minimum error between *in vitro* and *in silico* CV profiles (Figure 5E). We iterated this procedure 50 times and then we provided the mean and the standard deviation for the N_RRP_ that correspond with the smallest error.

### Statistical Analysis

Mean values for the EPSP amplitudes, the CVs and the synaptic parameters were expressed as their respective mean ± their standard deviation. Differences between distributions were measured using the Kruskal-Wallis test which shows a significant difference when *p* < 0.05. In order to compare two dimensional data sets (Figures 1F, 6F) we used the cross validated Kolmogorov-Smirnov test for two-dimensional data that shows significant differences when *p* < 0.2 (Press and Teukolsky, 1988). In order to test the goodness of fit for the fitting of the synaptic parameters we ran a Kolmogorov-Smirnov one sided test for three different distributions – beta, gamma and normal. Out of these three we chose the one with the highest p value and the smallest distance between the real and the expected distributions.

## Results

### Motivation for Implementing MVR in the Model

To reproduce the synaptic release variability observed *in vitro*, we began by implementing UVR at all synaptic contacts in the neocortical microcircuit model. As a result, the synaptic responses *in silico* were highly variable in comparison against biological data. In order to further investigate the potential causes for this difference in response variability, we undertook whole-cell recordings *in vitro* from 33 pairs of connected L5_TTPCs cells (Figure 1A, top) and computed the amplitude and the CV of the amplitudes for each EPSP. Figure 1 shows exemplar traces *in vitro* (Figure 1A, left in red) and *in silico* (Figure 1B, right in blue). As it was expected differences in the shape, amplitude and noise of the mean traces can be seen. The *in vitro* trace in red has a higher amplitude than the *in silico* in blue. Is also visible that the shape of the *in silico* mean trace is noisier than the *in vitro*, reflecting larger variability between protocol repetitions.

Next, we compared the distribution profiles of the first EPSP amplitude for the entire *in vitro* dataset (*n* = 33) and a subset of *in silico* connections (*n* = 100). Performing the Kruskal-Wallis test on the distributions of the first EPSP amplitude (Figure 1C) between *in vitro* and *in silico* connections revealed no significant difference in the mean values of their distributions (1.46 ± 0.86 mV for *in vitro*; 1.17 ± 0.57 mV for *in silico*; *p* = 0.15). However, a Kruskal-Wallis test between the distributions of the CV for the first EPSP amplitude (Figure 1E) revealed a significant difference in the mean values between *in vitro* and *in silico* connections (mean CV values: 0.38 ± 0.21 for *in vitro*; 0.45 ± 0.11 for *in silico*; *p* = 0.0092). Consequently, computing the CV profile for the EPSP amplitudes for every stimulus in a train showed a significant difference between *in vitro* and *in silico* data sets (Figure 1D; *p* < 10^–9^). The distributions (Figures 1C,E) were normalized to the respecting sample size such that the sum of products of width and height of each column was equal to the total count of connections (33 for *in vitro*, 100 for *in silico*). This difference was further corroborated through a Kolmogorov-Smirnov test for two-dimensional data (Press and Teukolsky, 1988), which also showed a significant difference between the first EPSP amplitude against the CV of the first EPSP amplitude for *in vitro* and *in silico* datasets (Figure 1F; *p* = 0.0022).

This striking difference motivated us to implement the MVR hypothesis, which is known to provide enhance the dynamic range of synapses through higher variability (Wang et al., 2006; Brémaud et al., 2007).

### Validating the Method

**FIGURE 2:**
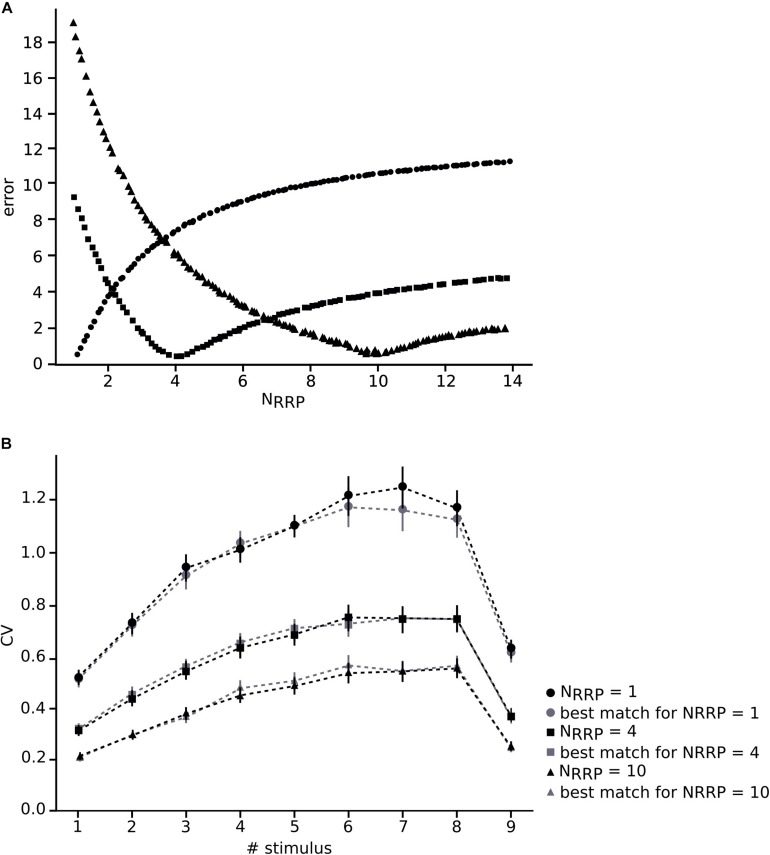
Validating the method. **(A)** Varying N_RRP_ against error for the different *in silico* data sets around the appropriate corresponding N_RRP_ (dots, N_RRP_ = 1; squares, N_RRP_ = 4; triangles, N_RRP_ = 10). **(B)** Mean CV profiles of the three different *in silico* data sets (black) and the simulations (gray). Dots, squares and triangles represent the mean while the error bars show the standard deviation.

Before applying our method to an *in vitro* data collection, we wanted to ensure that we were able to achieve the correct N_RRP_ value by using our procedure. For this purpose, we built 3 *in silico* data sets with different averaged N_RRP__s_ with mean values around 1, 4 and 10, each of them composed of 30 L5TTPC connections, similarly to the number of connections that is possible to obtain from *in vitro* experiments. Next, we simulated 100 *in silico* L5_TTPC connections with average N_RRP_ values ranging from 1 to 14 (see section “*In silico* Experiments: The Cortical Microcircuit”) and compared them against each CV profile computed through the JKK approach obtained from each of the *in silico* data sets (Figure 2B). Each *in silico* data set and the corresponding simulations consisted of different pairs of L5_TTPC connections.

In this manner, we obtained N_RRP__s_ that characterized each of the three different *in silico* data sets. We computed a minimum error around the correct value of each *in silico* data set (1, 4 and 10) which corresponding N_RRP__s_ were 1.01 ± 0.10, 4.07 ± 0.30 and 9.85 ± 0.45, obtaining as results N_RRP_ = 1.10 ± 0.31 (dots), N_RRP_ = 4.11 ± 1.75 (squares) and N_RRP_ = 10.71 ± 3.21 (triangles), respectively (Figure 2). By comparing the CV profiles between the *in silico* data sets (black) and the simulations (gray) (Figure 2B) we found that they were not significantly different (*p* > 0.4), which validated the efficacy of our method.

### Extracting Values for the TM-Model and Noise Calibration

**FIGURE 3:**
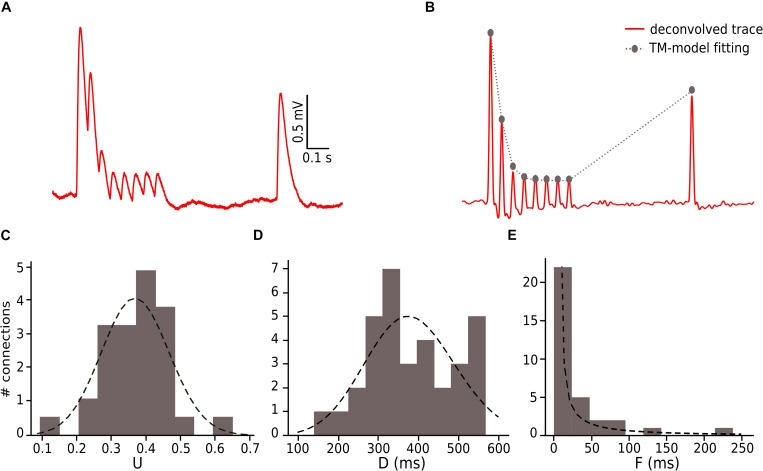
Fitting *in vitro* data to the TM-model. **(A)** Example of an *in vitro* mean voltage trace of L5_TTPC connection. **(B)** Corresponding deconvolved voltage trace (red) with the fit to the deterministic TM-model (gray). **(C)** Distribution of the probability of release parameter (U), **(D)** distribution of the time to recovery from depression (D) and **(E)** distribution of the time to recovery from facilitation (F). Values obtained from the fitting to the TM-model of 33 *in vitro* connections.

**FIGURE 4:**
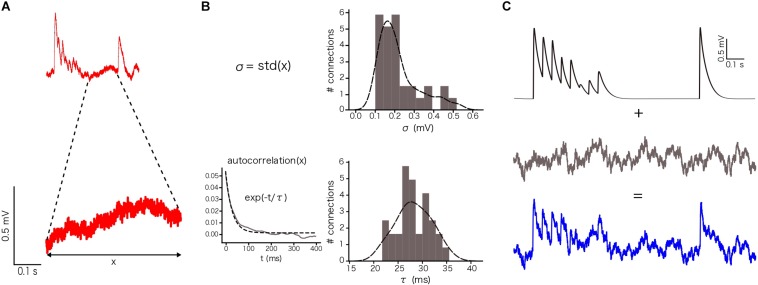
Noise calibration. **(A)** Example of an *in vitro* single protocol repetition (top). Zoom over 400 ms segment used to compute the parameters for noise calibration (bottom). **(B)** Distribution of σ (up) and τ (bottom). σ was computed as the standard deviation of the voltage segment. τ was computed by fitting the voltage segment autocorrelation to an exponential. The distributions show the mean values for the 33 *in vitro* connections. **(C)** (up) Single *in silico* trace without noise, (middle) OU-process generated to be added to the single *in silico* trace and (bottom) the noisy single protocol repetition that is the result of adding the previous two traces.

To enable comparison between the *in vitro* and the *in silico* experiments, we used the TM synapse model to extract the U, D, and F parameters from the *in vitro* dataset (see Materials and Methods). These parameters were obtained by the deconvolution of each *in vitro* averaged trace (Figure 3B) to extract the values of the peaks from the same voltage level. This resulted in three distributions, one each for U, D, and F, respectively. For U we obtained a normal distribution (goodness of fit: *p* = 0.92; *D* = 0.097) with a mean value of 0.38 ± 0.1 (Figure 3C), D fitted a gamma distribution (*p* = 0.81; *D* = 0.11) with a mean value of 365.6 ± 100.15 ms (Figure 3D) and F was also fitted to a gamma distribution (*p* = 0.1; *D* = 0.21) with mean 25.71 ± 45.87 ms (Figure 3E). These values were similar to the values found in previous studies (Tsodyks and Markram, 1997; Wang et al., 2002). As the next step we estimated the g_max_ for connections. We simulated *in silico* connections by tuning an initial g_ma__x_ value until the first EPSP amplitude matched experimental measurements. The resulting g_max_ was 1.54 ± 1.20 nS, which is consistent with previous estimates (Markram et al., 1997, 2015; Ramaswamy and Markram, 2015; Ramaswamy et al., 2015), and enabled the *in silico* reproduction of synaptic physiology between L5_TTPCs connections. We also further calibrated the membrane voltage noise parameter by implementing an OU-process on the *in vitro* dataset (see “Materials and Methods”) to obtain σ = 0.22 ± 0.10 mV (Figure 4B, top) and τ = 28.2 ± 3.5 ms (Figure 4B, bottom). Thus, by prescribing U, D, F and g_max_ parameters, and adding a synthetic membrane voltage noise to each simulated *in silico* connection we captured the biologically observed synaptic variability in L5_TTPC connections.

### Optimizing N_RRP_ for L5_TTPC Connections

**FIGURE 5:**
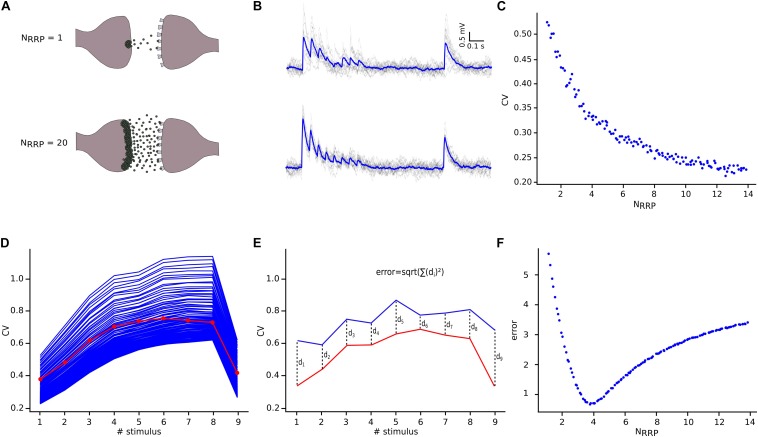
NRRP computation. **(A)** Illustration showing one synaptic connection releasing neuro transmitters from only one vesicle (top) and the same synaptic connection releasing neurotransmitters from twenty vesicles (bottom). **(B)** The corresponding effect of releasing neurotransmitters from one (top) or from twenty (bottom) vesicles reflected on the variability and shape of the *in silico* traces. The mean voltage traces are painted in blue while each protocol repetition is represented in gray. **(C)** Diagram showing the effect of N_RRP_ over the CV. **(D)** Mean CV profile for the *in vitro* (red) and all the *in silico* connections with different N_RRP_ values. **(E)** Diagram explaining the mean square distance computation. **(F)** N_RRP_ against error, showed a clear minimum around the value obtained for this specific connection.

Having defined the core synaptic parameter set, we next simulated *in silico* L5_TTPC connections as described before, although now we compared them against the CV_JKK_ computed from the *in vitro* data set. We observed a specific relationship between N_RRP_ and the CV for L5_TTPC connections (Figure 5C) that fits the power law with amplitude = 0.55 ± 0.015 and index = −0.39 ± 0.032. Initially, we observed that the CV for the first EPSP amplitude was higher when N_RRP_ was smaller. Therefore, for UVR-like connections the variability between individual sweeps is larger than for MVR-like connections. This result is in agreement with previous studies (Wang et al., 2006; Brémaud et al., 2007) and is also reflected in the simulated *in silico* connections with N_RRP_ = 1 (Figures 5A,B, top) and N_RRP_ = 20 (Figures 5A,B, bottom) to illustrate how the variability and voltage profile of EPSPs changes with the number of released vesicles.

In order to determine N_RRP_, we next computed the CV profiles of the *in silico* connections simulated with different N_RRP__s_ and measured their mean square distance (Figure 5E) in comparison against the *in vitro* CV profile (Figure 5D). We found that for L5_TTPC connections the minimum error was obtained with N_RRP_ = 3.78 ± 1.65 (Figure 5F), which demonstrates that our predictions of MVR for these connections is consistent with previous reports (Loebel et al., 2009; Rollenhagen et al., 2018).

### Implementing MVR Improved the Variability of the Synapses in the Model

**FIGURE 6:**
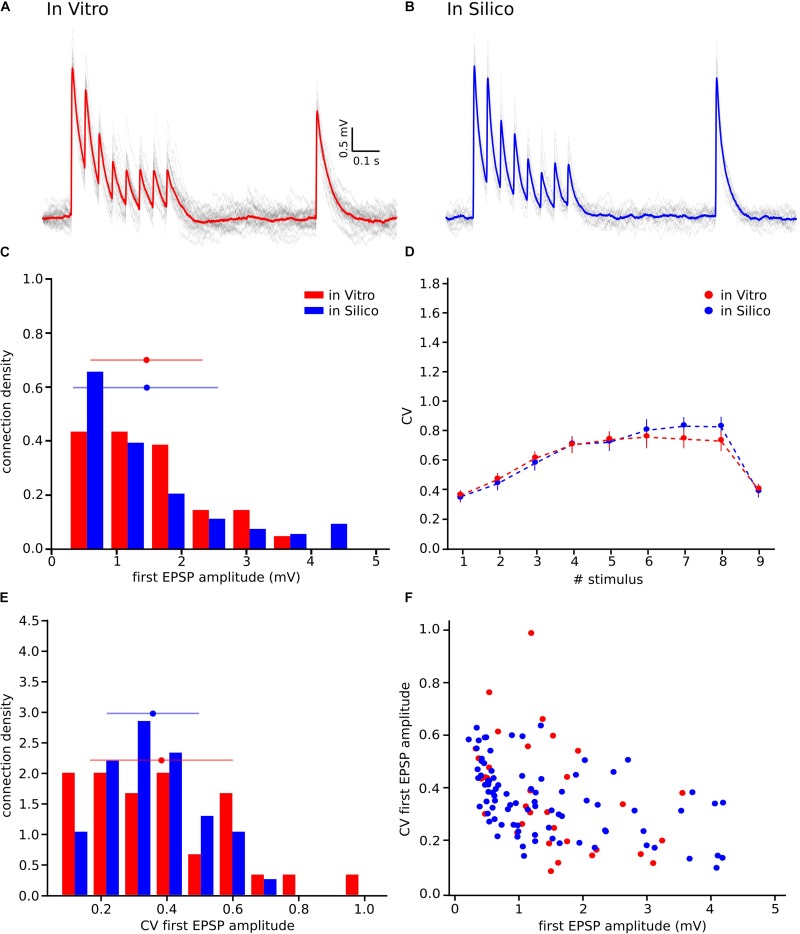
Releasing multiple vesicles improved the variability of the model. **(A)** *In vitro* mean voltage trace (red) of 20 protocol repetitions (gray) (same as in Figure 1A). **(B)** *In silico* mean voltage trace (blue) of 20 protocol repetitions (gray). **(C)** Distribution of the first EPSP amplitude for *in vitro* (red) and for the *in silico* (blue) experiments. **(D)** Mean CV profiles for the *in vitro* (red) and the *in silico* (blue) experiments. **(E)** CV Distribution of the first EPSP amplitude for *in vitro* (red) and the *in silico* (blue) data sets. **(F)** Raster plot of the first EPSP amplitude against the CV of the first EPSP amplitude for *in vitro* (red) and *in silico* (blue) experiments. All the *in silico* experiments are done with the N_RRP_ value that produces the minimum error. In the distributions and the CV profile, dots represent the mean and vertical and horizontal bars represent the standard deviation of all the experiments.

We next sought to test if our hypothesis of MVR between L5_TTPCs could better explain variability in experimental as against UVR (Figure 1). Therefore, we computed the distributions for the first EPSP amplitude, the CV of the first EPSP amplitude, and the CV profile of the EPSP amplitudes for all stimuli in a train. We found that the shape and the amplitude a randomly chosen *in silico* connection mediated by MVR (Figure 6B) was similar to a randomly chosen *in vitro* trace (Figure 6A), in contrast to an *in silico* connection mediated by UVR discussed before (see Motivation for implementing MVR in the model; Figure 1B, bottom). The CV profile for the EPSPs of all MVR *in silico* connections (Figure 6D, blue) also closely matched the *in vitro* dataset (red) as against UVR *in silico* connections (Figure 1D). Although our model has a slightly higher CV for the 6th, 7th, and 8th EPSPs, the Kruskal-Wallis test showed no significant differences between both CV profiles for any of the EPSPs (*p* = 0.89, *p* = 0.52, *p* = 0.42, respectively), demonstrating that the MVR hypothesis improved the synaptic variability of *in silico* connections.

Further results, shown in the distributions for the first EPSP amplitude (Figure 6C) and for the CV of the first EPSP amplitude (Figure 6E) corroborated the fact that MVR explained the experimentally observed variability better in contrast to UVR. The mean value of both MVR distributions was statistically insignificant compared against experimental data (mean EPSP values: 1.46 ± 0.86 mV for *in vitro*; 1.46 ± 0.95 mV for *in silico*; *p* = 0.69) (mean CV values: 0.38 ± 0.21 for *in vitro*; 0.35 ± 0.13 for *in silico*; *p* = 0.86). The distributions (Figures 6C,E) were normalized to the respective sample size such that the sum of products of width and height of each column is equal to the total count (33 for *in vitro*, 85 for *in silico*). In addition, a Kolmogorov-Smirnov test showed no significant difference between the first EPSP amplitude against the CV of the first EPSP amplitude for *in vitro* and *in silico* connections (*p* = 0.29) (Figure 6F), conclusively demonstrating that both data sets could, in principle, come from the same population.

### N_RRP_ Prediction for Other Cell-Type-Specific Connections

We extended this method to other cell-type-specific connections predicted in the neocortical tissue model (Markram et al., 2015; Ramaswamy et al., 2015; Reimann et al., 2015) and also independently characterized by other groups (Feldmeyer et al., 2002, 2005, 2006; Wang et al., 2002). Specifically, we computed the amplitudes and CVs of first PSP amplitudes from these published studies due to lack of access to raw experimental data. Synaptic parameter specifications for the different connections in the model are described in the NMC portal (Ramaswamy et al., 2015).

**FIGURE 7:**
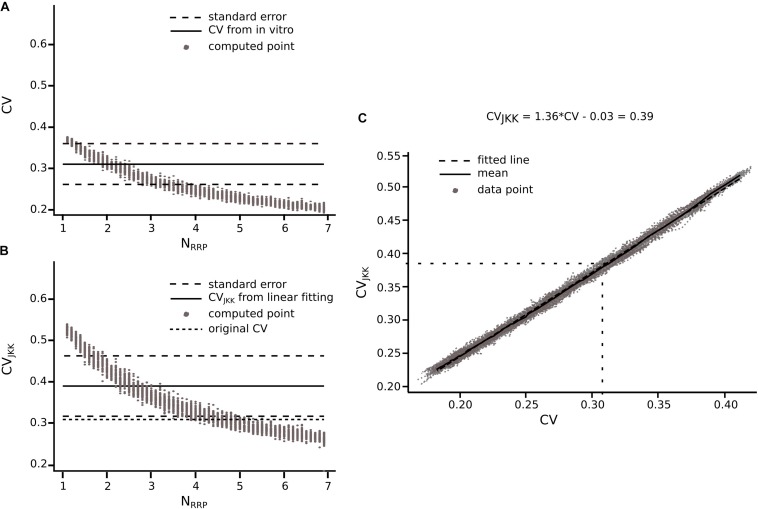
Extension of the method for connections reported in literature. Transformation from CV to CV_JKK_ using L5_TTPC connection as example **(A)** CV computed for different N_RRP_. Solid black line represents the CV computed for the *in vitro* data. Dotted black lines represent the standard error of the CV. **(B)** CV_JKK_ computed for different N_RRP_. Solid black line represents the CV_JKK_ obtained from the lineal fitting on **C**. Dotted black lines represent the standard error for this CV_JKK._ Short dotted black line represents the original CV found in literature. **(C)** CV to CV_JKK_ transformation. Solid black line represents the mean of the 50 iterations and dotted black line represent the linear fitting which equation is at the top of the plot. In **(A,B)** the gray dots show the 50 iterations from which we extract the best N_RRP_ as the one corresponding with the closest CV.

Before computing the CV for different cell-type-specific synaptic connections obtained from the literature, we had to take into account that they were not necessarily computed using the JKK bootstrapping approach. Our previous analyses demonstrate that the CV of the first EPSP computed through the JKK method has a slightly larger value than the CV computed analytically. In the case of L5_TTPC connections the CV_JKK_ was 0.38 ± 0.21 as against the analytical CV of 0.31 ± 0.14 for the *in vitro* data set but the N_RRP__s_ computed after 50 iterations in both cases were mostly similar (N_RRP_ without JKK = 2.41 ± 1.08 and N_RRP_ with JKK = 2.73 ± 1.22; *p* = 0.94; Figures 7A,B, respectively). This N_RRP_ obtained by comparing the *in vitro* and the *in silico* CVs for only the first EPSP is smaller than the previous N_RRP_ obtained by comparing the CV for all the EPSPs, but as revealed in the previous analysis we did not match the exact CV value for the 1st pulse, although there were no significant difference.

Knowing that the JKK bootstrapping method provided a more accurate method to compute EPSP amplitudes, we applied a transformation from CV to CV_JKK_ (Figure 7C). First, we computed the CV of the first EPSP amplitude without (Figure 7A) and with the JKK (Figure 7B) method. Second, we represented both CVs in the same plot for the different N_RRP_ values and we performed a linear fit to the mean of 50 repetitions (Figure 7C). Next, we determined the corresponding CV value computed with the JKK approach (Figure 7B), for this connection (L5_TTPC) we obtained CV_JKK_ = 0.39 ± 0.15 with a correspondent N_RRP_ = 2.84 ± 1.34. We did that for every connection for which we could find data in the literature and our simulation matched the variability (Table 1).

**TABLE 1:** Results for connections reported in literature.

**Connection type**	**Literature data**	**Jack-Knife conversion**	**Prediction**
	**CV**	**CV**	**N_RRP_**

**L23_NBC_LBC-L23_PC**	0.40 ± 0.09 (Wang et al., 2002)	0.38 ± 0.21	1.96 ± 0.98
**L23_PC-L23_PC**	0.33 ± 0.18 (Feldmeyer et al., 2006)	0.48 ± 0.23	2.60 ± 1.28
**L4_SSC-L23_PC**	0.27 ± 0.13 (Feldmeyer et al., 2002)	0.37 ± 0.09	1.81 ± 0.37
**L4_SSC-L5_TPC:C**	0.33 ± 0.20 (Feldmeyer et al., 2005)	0.46 ± 0.15	1.26 ± 0.50
**L5_TTPC-L5_SBC**	0.32 ± 0.08 (Wang et al., 2002)	0.34 ± 0.16	1.82 ± 0.90
**L5_TTPC-L5_TTPC**	0.31 ± 0.14 (Measured in this study)	0.39 ± 0.15	2.84 ± 1.34

*Table summarizing the CV_*JKK*_ computed for other five cell connections through the collection of data from literature and applying the JKK conversion explained in Figure 6. For the L5_TTPC connection we used the CV computed in this work from our *in vitro* data set. L23_NBC_LBC: layer 2 and 3 nest and large basket cells; L23_PC: pyramidal cells in layer 2 and 3; L4_SSC: layer 4 spiny stellate cells; L5_TPC:C: thick tuft pyramidal cells that receive projections from thalamus; L5_SBC: small basket cells from layer 5.*

The generalized results to five different cell-type-specific connections are summarized in Table 1. We further predict that for connections between layer 4 spiny stellate (L4_SSC) and slender-tufted layer 5 pyramidal cell connections that project across the corpus callosum (L5_TPC:C), synaptic release is mediated by UVR (see Table 1; N_RRP_ = 1.26 ± 0.50), while for the remainder of connections the predicted N_RRP_ is between 2 to 3 (see Table 1; N_RRP_ = 2.60 ± 1.28 for L23_PC-L23_PC; N_RRP_ = 1.96 ± 0.98 for L23_NBC_LBC-L23_PC; N_RRP_ = 1.81 ± 0.37 for L4_SSC-L23_PC and N_RRP_ = 1.82 ± 0.90 for L5_TTPC-L5_SBC).

Our results predict that synaptic release at most connections in the neocortex are more likely mediated by MVR rather than UVR, supporting the idea that the release of multiple vesicles enhances the response variability of neocortical synapses and augments information transmission.

## Discussion

In this work we computed the N_RRP_ building on the previous work of Loebel et al. (2009) but extended it to all individual synaptic contacts in a connection. Our approach is based on the comparison of the amplitudes and CV of EPSPs between cell-type-specific *in vitro* and *in silico* connections with different N_RRP_ values within the framework of a large-scale, data driven tissue level model of juvenile rat neocortical microcircuitry (Markram et al., 2015). The CV of the amplitude distributions reliably reflects the concentration of neurotransmitter in the synaptic cleft and for the postsynaptic receptor occupancy (Faber and Korn, 1991; Auger and Marty, 2000; Neishabouri and Faisal, 2014). For example, a large quantity of presynaptic neurotransmitter release would give rise to a high amplitude EPSP. However, a large fraction of receptors would be occupied as well and consequently it would be more difficult to generate a second EPSP if more neurotransmitter is released. Thus, it is possible to measure the variability of the EPSP amplitude considering that high variability represents a small number of released vesicles.

### UVR Cannot Reproduce the Variability Observed Into the Biological Data

Our analysis demonstrates that the UVR hypothesis cannot reproduce the variability observed on the *in vitro* traces, in fact the CV profile for the *in silico* experiments is significantly larger, although the first EPSP amplitude is not statistically different. This result suggests that the MVR hypothesis could be more relevant to explain the response variability in neocortical synapses. On the one hand, this idea differs from previous studies (Redman, 1990; Gulyás et al., 1993; Murphy et al., 2004), which claim that at each active zone in a synapse only one vesicle could be released, suggesting that the biological variability may come from changes in the quantal size. On the other hand, more recent studies validate our MVR hypothesis that better explains biological variability (Brémaud et al., 2007; Loebel et al., 2009; Hardingham et al., 2010; Huang et al., 2010; Rudolph et al., 2015). This discrepancy could be partly attributed to the fact that the studies validating the UVR hypothesis were undertaken in brain regions other than the neocortex, with different experimental protocols, across different species and cell-types.

Before obtaining evidence, which supports the MVR hypothesis, we extracted a core set of synaptic important parameters from an *in vitro* dataset obtained from L5_TTPCs. First, we computed the parameters pertaining to a deterministic model of short-term synaptic depression (Tsodyks and Markram, 1997). To this end, we had to select only those connections whose 1st EPSP amplitude was within the range of the *in vitro* data set and apply the deconvolution for computing the peaks. Then we introduced the peak values on a GA that calculated the synaptic parameters. The values obtained were similar to values found in previous researches (Tsodyks and Markram, 1997; Wang et al., 2006). Second, we calibrated the synaptic noise which represented the synaptic trial-to-trial variability. Many studies support the idea that background synaptic noise is not merely “noise,” but an addition of various meaningful mechanisms as channels and receptors dynamics (Azouz and Gray, 1999; Faisal et al., 2008). Synaptic noise is also thought to arise from the spontaneous fusion and release of vesicle (Fatt and Katz, 1950). This noise could not only influence the synaptic variability, but also the transmission of information (Jacobson et al., 2005). Thus, while some studies do not support our hypothesis of the contribution of the number of vesicles in synaptic noise (Mackenzie et al., 2000), several others (Korn et al., 1993; Franks et al., 2003; Faisal et al., 2008; Pulido and Marty, 2017) inspired us to include additional synaptic noise in our model. Finally, we also validated our method by building three different *in silico* data sets where the mean N_RRP_ was set to 1, 4 and 10, respectively. Although the mean values obtained using the method were slightly larger, no significant differences were found, and therefore, we used the validated method with experimental data sets.

### L5_TTPC Synapses Are Driven by Multiple Vesicles

Increasing the N_RRP_ improved the variability of our model, resulting in synapses that more faithfully reproduced the experimentally observed physiology. Consequently, for synaptic connections between L5_TTPCs the predicted N_RRP_ was 3.78 ± 1.65 within a range of 1 to 9 vesicles. Synaptic connections between L5_TTPCs are mediated by about 4 to 8 contacts on average (Markram et al., 1997). We predict that the total number of release sites for pairs synaptic contact between L5_TTPC connections ranges between 4 to 72, which is consistent with two previous studies of that have estimated vesicles in L5_TTPC synaptic contacts to range from 2 to 30 docked vesicles (Rollenhagen and Lübke, 2006), and 7 to 170 vesicles (Loebel et al., 2009). Our predictions are also consistent with a recent study, which estimated that the number of readily releasable vesicles at individual synaptic contacts of L5B PCs ranged from 1.2 to 12.8 with an average of (5.40 ± 1.24) per contact (Rollenhagen et al., 2018). The estimated mean value is slightly larger than what we predict, which could be due to a difference in the developmental age and the cortical area. While our experimental data set was obtained in the non-barrel hind limb somatosensory cortex of juvenile rats, Rollenhagen et al. (2018) investigated synapses between L5B PCs in the barrel cortex of adult rats. Compared to cortical synapses, the neuromuscular junction and the Calyx of Held, which are extensively studied synaptic assemblies, also show MVR with about two, and three vesicles per active zone, respectively (Neher and Sakaba, 2008; Ruiz et al., 2011; Sakaba, 2018). These studies support the idea that MVR occurs in different brain areas within different ranges, suggesting that MVR may be important not only for reliable information transmission, but also a key mechanism for defining synaptic functionality. Is synaptic release in other cell-type-specific connections in the rat neocortex mediated by MVR?

We extended our method to predict the N_RRP_ for L5_TTPC synapses to other cell-type-specific connections in the neocortex reported in the literature. For five different cell-type-specific connections, we predict that the average N_RRP_ is between 2 and 3 (see Table 1). Although our predictions are inconsistent with some observations, for connections between L4_SSC and L23_PCs (Silver et al., 2003), they are comparable with other studies that support the notion of MVR as a fundamental property of intra and inter-laminar cortical synapses (Brémaud et al., 2007; Huang et al., 2010).

Due to lack of specific data, we extrapolated synaptic parameters measured in the superficial layers (Wang et al., 2002) to deeper layers, in particular for synaptic connections between L2/3 PCs and basket cells to their counterparts L5 to predict the N_RRP_. Our data-driven framework is designed in to integrate specific data sets as and when they become available to enable predictions on the N_RRP_ of cortical synapses.

Despite the occurrence of weak *in silico* synaptic connections between L5_TTPCs in the neocortical tissue model, the CV distribution has a lower mean because the subset of *in silico* connections that were sampled to reproduce experimental findings display high EPSP amplitudes. Previous work seems to suggest that weak synaptic connections are necessary to maintain synchronous activity in the cortex (Bruno and Sakmann, 2006; Ren et al., 2017). Therefore, future refinements of this approach should consider how weak connections could impact predictions of N_RRP_. It should be noted that other parameters relevant to predict the N_RRP_, such as g_max_ were determined indirectly in our study, which could impact our results. For instance, if g_max_ was underestimated, we would have had obtained a larger N_RRP_ by increasing its value considering the same CV. It is also known that other synaptic mechanisms such as the membrane fusion, receptor saturation, and vesicle recycling directly influence vesicle release (Stevens, 2003; Watanabe et al., 2013; Rizo and Xu, 2015; Rudolph et al., 2015). We propose that future work should consider all these synaptic factors to predict N_RRP_ for cortical connections.

In summary, we described an approach built upon previous work (Loebel et al., 2009) to predict the N_RRP_ per active synaptic contact for neocortical connections. By systematically comparing *in vitro* and *in silico* data on the CV of the EPSP amplitude CV, we could predict the N_RRP_. Our preliminary results suggest that MVR could serve as a fundamental mechanism in the brain to increase the dynamic range of synapses and their variability.

## Data Availability Statement

The raw data supporting the conclusions of this manuscript will be made available by the authors, without undue reservation, to any qualified researcher.

## Ethics Statement

All experiments were performed according to the Swiss national and institutional guidelines.

## Author Contributions

NB-Z developed and performed the data analysis and the *in silico* experiments, drafted the manuscript, and generated the figures. JR developed and performed the initial data analysis. GC developed and performed some data analysis and some *in silico* experiments. RP designed and performed the *in vitro* experiments. HM contributed to data interpretation and procured funding for the study. SR gathered data from the published literature, contributed to data interpretation, and drafted the manuscript. EM contributed to data interpretation and an initial draft of the manuscript. SR and EM jointly conceived and supervised the study.

## Conflict of Interest

The authors declare that the research was conducted in the absence of any commercial or financial relationships that could be construed as a potential conflict of interest.
